# Successful switch to ofatumumab after liver injury associated with ocrelizumab treatment in multiple sclerosis: a case report

**DOI:** 10.3389/fneur.2024.1363493

**Published:** 2024-02-29

**Authors:** Alice Mariottini, Alessandro Barilaro, Antonio Lotti, Fabio Marra, Luca Massacesi

**Affiliations:** ^1^Department of Neurosciences, Drug and Child Health, University of Florence, Florence, Italy; ^2^Department of Neurology 2 and Tuscan Region Multiple Sclerosis Referral Centre, Careggi University Hospital, Florence, Italy; ^3^Dipartimento di Medicina Sperimentale e Clinica, University of Florence, Florence, Italy

**Keywords:** case report, liver injury, ocrelizumab, ofatumumab, multiple sclerosis, monoclonal antibody, B-cell depletion

## Abstract

Drug-induced liver injury (DILI) is a potential adverse event of disease-modifying therapies (DMTs) for the treatment of multiple sclerosis (MS), as well as of methylprednisolone pulsed therapy used in case of MS relapse. DILI may be induced by different mechanisms, including idiosyncratic reaction, autoimmune hepatitis or viral reactivation. In patients receiving the humanized anti-CD20 monoclonal antibody (mAb) ocrelizumab, DILI has been rarely reported and was mostly associated with hepatitis B virus (HBV) reactivation. Here we present the case of a woman with highly active relapsing–remitting MS who had experienced two episodes of DILI while receiving different DMTs, and was successfully switched to ofatumumab, a fully human anti-CD20 mAb, after a further event associated with ocrelizumab treatment and unrelated to HBV reactivation. Despite sharing the mechanism of action, differences in structure, pharmacokinetic/pharmacodynamic profile, and use of ancillary drugs (only needed for ocrelizumab) may have accounted for the successful switch. To our knowledge, this is the first report of a successful switch from ocrelizumab to ofatumumab due to DILI. Ofatumumab may therefore represent a valid therapeutic option for patients experiencing DMTs- and ocrelizumab-induced liver injury, providing that HBV reactivation has been ruled out.

## 1 Introduction

Drug-induced liver injury (DILI) is a potential adverse event of disease-modifying therapies (DMTs) for the treatment of multiple sclerosis (MS), an autoimmune demyelinating and degenerative disease of the central nervous system (1, 2). In this context, severe hepatic failure is a rare event, and drug discontinuation rate due to hepatic injury is generally <1% (1). Nonetheless, a signal of disproportionate reporting of DILI events was observed for several DMTs in a pharmacovigilance study based on the Food and Drug Administration (FDA) Adverse Events Reporting System (FAERS) (3). Of note, DILI has been associated also with pulsed intravenous methylprednisolone (IVMP) therapy used for the treatment of MS relapse (4).

Anti-CD20 monoclonal antibodies (mAb) are increasingly used for the treatment of MS (5). The humanized mAb ocrelizumab, approved in 2017–2018 by the FDA and European Medicines Agency (EMA) for the treatment of relapsing MS and primary progressive MS (6), has been rarely associated with alterations of the hepatic profile. More recently (2020–2021), ofatumumab, a fully human anti-CD20 mAb, was approved by the Agencies for relapsing MS (7). These two mAbs have the same mechanism of action but are administered via different routes (intravenous [IV] for ocrelizumab and subcutaneous [SC] for ofatumumab) and exhibit different pharmacokinetic/pharmacodynamic profiles (8), summarized in Table 1.

**Table 1:** Pharmacodynamic and pharmacokinetic profiles of ocrelizumab and ofatumumab.

	Ocrelizumab	Ofatumumab
Structure	Recombinant humanized monoclonal anti-CD20 IgG1 antibody (molecular mass of approximately 145 kD)	Fully human anti-CD20 monoclonal IgG1 antibody (theoretical average molecular weight of 145 kDa).
Administration route	Intravenous	Subcutaneous
Dosing	600 mg IV every six months^a^	20 mg SC every four weeks^b^
Ancillary drugs	100 mg methylprednisolone (or equivalent) Antihistamines	Not required
Half-life	26 days	16 days
Elimination	not directly studied, as antibodies are cleared principally by catabolism (i.e., breakdown into peptides and amino acids).	target-mediated route (related to binding to B cells) and target-independent route (non-specific endocytosis followed by intracellular catabolism)
Time to CD20^+^ cells depletion	By 14 days	By 14 days
Median time to CD20^+^ B cells recovery	72 weeks	24.6 weeks

Data derived from refs. (6, 7). ^a^First dose administered as two 300 mg IV doses two weeks apart. ^b^Maintenance after a loading dose consisting of three 20 mg SC weekly administration.

Therapeutic management of patients who have experienced DILI may be challenging. We report the case of a woman with highly active relapsing–remitting (RR) MS who was successfully switched to ofatumumab after having experienced multiple episodes of DILI, the latest occurring during ocrelizumab treatment, and unrelated to hepatitis B virus (HBV) reactivation. To our knowledge, this is the first report of a successful switch from ocrelizumab to ofatumumab due to liver toxicity.

## 2 Case presentation

A 57-year-old woman was diagnosed with RRMS when she was 29 years old. At that time, she experienced an episode of numbness in her left upper limb. Her past medical history was unremarkable except for vitiligo. No familiar history of MS or other autoimmune disorders was reported. Magnetic resonance imaging (MRI) showed multiple brain hyperintense T2 lesions involving areas typical for MS, and one spinal cord lesion. Oligoclonal bands were detected in the cerebrospinal fluid, and the diagnostic work-up did not show any red-flags of better explanation. Clinically definite MS was diagnosed a few months later after the occurrence of optic neuritis and increased lesion load at MRI. Starting from 1999, she was sequentially treated with interferon beta-1a (IFN), azathioprine, glatiramer-acetate, and then switched in 2013 to IFN 44 mcg thrice a week. The treatment with IFN 44 was uneventful until February 2016, when the patient complained of marked fatigue and jaundice. Blood chemistries showed a marked elevation of serum aspartate aminotransferase (AST, 819 UI/L; upper limit of normal [ULN] 41 UI/L), serum alanine aminotransferase (ALT, 498 UI/L; ULN 50 UI/L), and gamma-glutamyl transpeptidase (GGT, 302 UI/L; ULN 38 UI/L), with mild elevation of serum alkaline phosphatase (ALP, 157 UI/L; ULN 126 UI/L) and serum total bilirubin (2.5 mg/dL; ULN 1.2 mg/dL) with prevalent increase of the conjugated fraction. IFN was discontinued and two months later, after normalization of liver enzymes, she was switched to fingolimod. Unfortunately, nine months later (February 2017) fingolimod was discontinued due to a diagnosis of breast cancer, which was successfully treated with surgery, followed by tamoxifen treatment for the subsequent 5 years. In August 2017, the patient was started on glatiramer-acetate, but this was discontinued a few months later due to recurrent allergic reactions to the drug and a grade 2 increase in serum aminotransferases. Liver enzymes normalized spontaneously within one month from DMT discontinuation, and the event was attributed to the possible combined hepatic toxicity of glatiramer-acetate and tamoxifen. A transient, mild elevation in serum aminotransferase was again observed a few weeks later, shortly after a course of IVMP for MS relapse (balance issues and lower limbs hypoesthesia with T5 level). She was then switched to dimethyl-fumarate and in June 2020, after a further course of IVMP for MS relapse (dizziness and left-sided facial numbness), she experienced a remarkable elevation in liver enzymes requiring hospital admission. The patient was discharged with a diagnosis of probable DILI associated with treatment with dimethyl-fumarate/high-dose IVMP.

**Figure 1:**
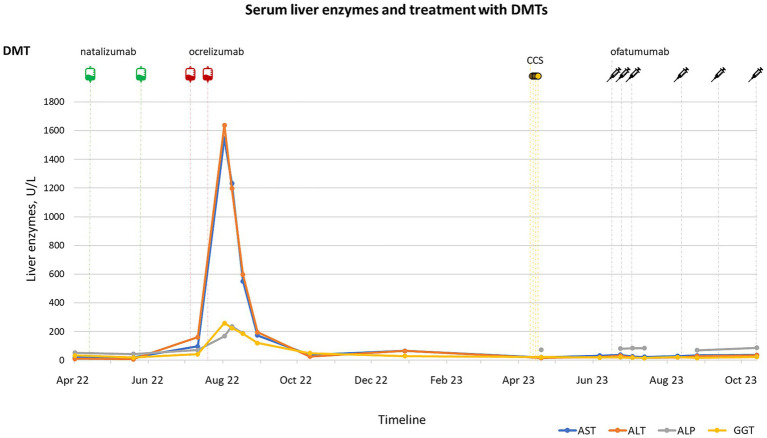
Timeline of disease-modifying treatments (DMTs) and dexamethasone (CCS) administration (upper panel) and serum liver tests (lower panel) during the latest episode of DILI experienced by the patient. A mild elevation in serum transaminase [serum alanine aminotransferase (ALT), and serum aspartate aminotransferase (AST)] was observed shortly after the first administration of ocrelizumab 300 mg IV, followed by a severe elevation in transaminase, gamma-glutamyl transpeptidase (GGT) and serum alkaline phosphatase (ALP) detected two weeks after the second dose. The levels of liver enzymes returned below the upper limit of normal (ULN) within two months, without the administration of any corticosteroid treatment. No further elevations were observed during the follow-up, including after ofatumumab start.

Due to recurrence of MS activity, the patient was started on natalizumab in October 2020. She received 18 administrations of natalizumab with clinical and MRI stabilization, in the absence of any elevations in serum liver enzymes, which were tested monthly. In May 2022, the drug was discontinued due to JCV positivity (index 3.56), and in July 2022 the patient was switched to ocrelizumab, after having received an abdominal ultrasound which did not show any abnormal liver findings. Six days after the first IV infusion of ocrelizumab 300 mg, a mild elevation in AST (within twofold ULN) and ALT (within threefold ULN) was observed, while ALP, GGT and bilirubin were all within normal ranges (Figure 1). One week after the second administration of ocrelizumab 300 mg IV, the patient complained of nausea, increasing fatigue and anorexia. Blood tests showed a marked increase in liver enzymes with a clear hepatocellular pattern (AST 1234 U/L, ALT 1198 U/L, ALP 236 U/L, GGT 224 U/L, bilirubin 4.5 mg/dL with 2.8 mg/dL conjugated; Figure 1). Anti-mitochondrial, anti-nuclear and anti-smooth muscle autoantibodies were all negative, as well as cytomegalovirus (CMV) and Epstein–Barr virus (EBV) DNA, anti-toxoplasma antibodies, HBsAg, anti-HBV antibodies and HBV DNA, which had tested negative also before ocrelizumab start. She only received IV hydration and liver enzymes gradually decreased, reaching values within the normal range in two months.

An abdominal ultrasound performed in December 2022 showed patchy echogenicity of the liver, suggestive of marked inhomogeneity of its parenchymal architecture, consistent with chronic liver disease. After consultation with her treating hepatologist, the prosecution of treatment with ocrelizumab was not recommended and the second dose scheduled for February 2023 was not administered. MS was clinically and radiologically stable up to April 2023 (brain MRI performed in June 2022, i.e., before starting ocrelizumab, and 3 months after the first dose of ocrelizumab were both unchanged compared to the previous examination taken in November 2021).

In April 2023, when B cells were still depleted, she experienced a spinal cord relapse (severe balance issues preventing her from walking unaided); at that time, the patient was no more taking tamoxifen (discontinued in April 2022). As, in the past, the administration of IVMP was followed by derangement of liver enzymes, intramuscular dexamethasone was chosen over IVMP to treat the relapse. After dexamethasone treatment, a good recovery from MS relapse was observed, and no elevation in serum liver enzymes was detected. A further abdomen ultrasound (May 2023) showed the persistence of an inhomogeneous liver structure with a pseudonodular pattern, and normal stiffness at elastography. Further investigation with a dedicated MRI was suggested, which did not show any abnormalities, nor pathological areas of diffusion restriction.

After further consultation with the Hospital hepatologist, ofatumumab was started in June 2023, with three weekly SC doses of 20 mg over the first month (loading dose), followed by a monthly dose of 20 mg, with close monitoring of liver enzymes. At the time of writing, the patient has been treated with ofatumumab for five months, and no elevation in liver enzymes has been observed. No further relapses were reported, and a brain MRI performed in October 2023 showed no new lesions compared to the previous scan taken in September 2022. Adherence to ofatumumab treatment was confirmed by persistent B cell depletion at monitoring blood tests, starting from July 2023. At month 3 after ofatumumab start, the patient’s satisfaction with ofatumumab treatment was explored using the treatment satisfaction questionnaire of medication (TSQM) v1.4 (9), showing an 81% satisfaction rate.

## 3 Discussion

Pathogenetic mechanisms of DILI may be heterogeneous and include idiosyncratic reactions, trigger of autoimmune hepatitis (AIH) and viral reactivation (10). Differential diagnosis between DILI and AIH may be challenging, especially in patients with a pre-existing autoimmune disorder like MS, and both DILI and AIH have been described in association with DMTs and IVMP pulsed therapy (10). In our patient, liver injury was characterized by a hepatocellular pattern, autoantibodies were negative, and it resolved spontaneously after drug withdrawal. In our opinion, these findings overall suggest that its pathogenesis was plausibly idiosyncratic (11), although we lack histopathological assessment as liver biopsy was not performed due to the spontaneous resolution of injury after each episode.

A peculiarity of this case is that three distinct episodes of DILI (12) were observed over 7 years, occurring while receiving three different DMTs (IFN, dimethyl-fumarate and ocrelizumab), and showing a temporal association with IVMP in at least one occasion, when the patient was under tamoxifen treatment. IVMP pulsed therapy is known to be associated with liver injury. In a prospective observational study including 175 MS patients receiving IVMP (1,000 mg/day for 5 days) for disease activity, a 8.6% prevalence of liver injury was observed, being the injury severe (according to Hy’s law) in 2.5% of the cases (4).

In our patient, the association of IVMP with other drugs (IFN, tamoxifen or dimethyl-fumarate) may have increased the odds for DILI, as the use of IVMP alone was not followed by remarkable serum aminotransferase elevation. The potential hepatotoxicity of both IFN and dimethyl-fumarate is well established. In the LiverTox® database, which classifies drugs based on the number of published reports of convincingly documented idiosyncratic liver injury, IFN is assigned a likelihood score A (i.e., well-known cause of clinically apparent liver injury), whereas dimethyl-fumarate a score C (i.e., probable rare cause of clinically apparent liver injury) (13). Furthermore, IFN was associated with a signal of disproportionate reporting in a pharmacovigilance-based study (3). The use of concomitant therapies, including tamoxifen, may have contributed to the liver injury observed in our patient; accordingly, the use of concomitant drugs was reported in 47% of DILI cases from the FARES study (3).

Ocrelizumab is considered to be associated with a low likelihood of liver injury (category D, i.e., rare cause of clinically apparent liver injury), and this is probably related to HBV reactivation (13). With few exceptions (14–16), all the ocrelizumab-associated DILI events reported in the literature were indeed associated with HBV reactivation (1). B-cell depleting therapies may cause reactivation of hepatitis B through their immunosuppressive action, and HBV reactivation may, in turn, induce acute hepatocellular injury, potentially leading to acute liver failure (17). In our patient, serological markers of HBV infection were repeatedly negative, as it was HBV DNA, thus allowing us to rule out that the latest episode of DILI was associated with HBV reactivation. As low-dose IVMP (100 mg) is administered as an ancillary drug before ocrelizumab infusion, we cannot exclude that this might have contributed to the DILI event associated with ocrelizumab, also considering the previous history of IVMP-associated DILI (mostly observed in association with other treatments) (18).

The choice of switching the patient to ofatumumab was based mainly on the following reasons: (i) evidence of MS activity requiring treatment with a high-efficacy DMT; (ii) serum aminotransferase elevations are uncommon during ofatumumab therapy, and clinically apparent liver injury associated with this DMT has not been reported to date in the Livertox^®^ database (13); (iii) differences in structure, pharmacokinetic and pharmacodynamic between ocrelizumab and ofatumumab, including a different route of administration; and (iv) the lack of requirement for any ancillary drugs with ofatumumab. Treatment with alternative high-efficacy DMTs was also discussed. PML risk (index 3.54 plus previous exposure to immunosuppressive treatment) and patient’s convenience (she needs to travel for almost 3 h to get to the hospital) led to the exclusion of natalizumab, as it was deemed to have a detrimental impact on her quality of life, even if adopting an extended interval dosing. Sphingosine 1-phosphate modulators were also discussed, but their efficacy/safety profile was considered less favorable compared to that of CD20-depleting mAbs (18–20).

In our patient, the switch to ofatumumab was safe so far, as no further elevations in liver enzymes were observed over five months of treatment, and effective, without any new signs of MS activity.

The main limit of this case report is the short period of observation after ofatumumab start, and further follow-up is needed to confirm ofatumumab safety profile over long term, especially in case of concomitant administration of drugs with potential hepatotoxicity. Another limitation is the lack of histopathological liver assessment, as biopsy was not performed due to the spontaneous resolution of liver injury.

In conclusion, the present case report indicates that ofatumumab may be safely administered in patients who had experienced DILI associated with DMTs and specifically during ocrelizumab treatment, providing that HBV reactivation has been ruled out.

## 4 Patient perspective

The patient manifested a high rate of satisfaction with ofatumumab treatment, despite a tighter schedule of administration of ofatumumab compared to ocrelizumab and the SC route. The satisfaction was mainly driven by an optimal tolerability to the drug, including the lack of liver adverse events.

## Data availability statement

The datasets presented in this article are not readily available because of ethical and privacy restrictions. Requests to access the datasets should be directed to the corresponding author.

## Ethics statement

Ethical review and approval was not required for the study on human participants in accordance with the local legislation and institutional requirements. Written informed consent from the patients/participants or patients/participants' legal guardian/next of kin was not required to participate in this study in accordance with the national legislation and the institutional requirements. Written informed consent was obtained from the individual(s) for the publication of any potentially identifiable images or data included in this article.

## Author contributions

AM: Conceptualization, Data curation, Methodology, Visualization, Writing – original draft, Writing – review & editing. AB: Data curation, Writing – review & editing. AL: Data curation, Writing – review & editing. FM: Writing – review & editing. LM: Writing – review & editing.
